# Engaging Leadership and Its Implication for Work Engagement and Job Outcomes at the Individual and Team Level: A Multi-Level Longitudinal Study

**DOI:** 10.3390/ijerph17030776

**Published:** 2020-01-26

**Authors:** Vivi Gusrini Rahmadani, Wilmar B. Schaufeli, Jeroen Stouten, Zhenduo Zhang, Zulkarnain Zulkarnain

**Affiliations:** 1 Research Unit Occupational & Organizational Psychology and Professional Learning, KU Leuven, 3000 Leuven, Belgiumjeroen.stouten@kuleuven.be (J.S.); zhenduo.zhang@student.kuleuven.be (Z.Z.); 2 Fakultas Psikologi, Universitas Sumatera Utara, Medan 20155, Indonesia; zulkarnain3@usu.ac.id; 3 Department of Psychology, Utrecht University, 3508 Utrecht, The Netherlands

**Keywords:** engaging leadership, work engagement, job outcomes, multi-level, longitudinal

## Abstract

The current study investigates how supervisors’ engaging leadership, as perceived by their employees, increases employees’ job outcomes at the individual and team level, as mediated by (team) work engagement. Job outcome indicators at the team level are team performance, team learning, and team innovation; and at the individual level, job performance, employee learning, and innovative work behavior. The novel concept of engaging leadership is presented as the specific type of leadership to foster (team) work engagement. A multi-level longitudinal study is conducted among 224 blue collar employees nested in 54 teams in an Indonesian state-owned holding company in the agricultural industry using a one-year time lag. The findings show, as expected, that at the team level, engaging leadership at time 1 predicted team learning and team innovation (but not team performance) at time 2, via team work engagement at time 2. Additionally, an expected cross-level effect was observed from engaging leadership at the team level at time 1 predicting individual job performance (but not employee learning and innovative work behavior) at time 2, via team work engagement at time 2. Finally, an expected second cross-level effect was observed for engaging leadership at the team level at time 1, which predicted individual job performance, employee learning, and innovative work behavior at time 2, via work engagement at time 2.

## 1. Introduction

To achieve the critical goals of viability and sustained competitive advantage, organizations currently face global challenges [1] that require them to continuously perform, adapt, learn, and innovate in a rapidly changing environment [2]. To do so, the contributions of employees to organizational goals are increasingly important, especially in teams, as these are the building blocks for organizations [3] to address complex tasks by providing collaborative effort [4].

To ensure that teams are effective, they need support from their leaders. Leadership is a prominent antecedent in organizations that facilitates individual and collective (team) efforts to accomplish shared objectives and improve performance [5], as well as to adapt and innovate [6]. What is currently still missing is in-depth knowledge of intermediate processes to explain how leadership influences individuals, group processes and organizational effectiveness [7], including performance, learning, and innovation at the team and individual level. The current study focuses on such mediating motivational processes in which work engagement is assumed to play a key role. 

Work engagement (WE) refers to "a positive, fulfilling, work-related state of mind that is characterized by vigor, dedication, and absorption" [8] (p.74). Employees with high levels of work engagement display innovative behaviors at work [9,10] and are more creative [11]. Moreover, work engagement is positively related to business-unit performance [12]. In sum, work engagement benefits organizations at all levels, not only at the individual employee level but also at the team and business-unit level [13,14,15,16].

Whereas previous research mostly focused on individual work engagement, less is known about WE at the team level, which exists as a collective psychosocial phenomenon [17]. This team work engagement (TWE) is defined as a positive, fulfilling, and shared motivational emergent state that is characterized by team vigor, team dedication, and team absorption, which emerges from the interactions and shared experiences of members of a workgroup [18,19]. WE and TWE are related but distinct. TWE is experienced only when team members’ affective-motivational states converge, which is not necessarily always the case.

Based on the Job Demands–Resources (JD-R) Model [20], leaders manage and allocate job demands and job resources in ways as to increase employees’ levels of (team) work engagement. Hence, generally speaking, leaders will facilitate the motivational process that is postulated by the JD-R Model. This process assumes that job resources and challenging job demands are inherently motivating and that both will lead to a positive, affective-motivational state of fulfillment in employees that is known as (team) work engagement.

In the current research, we propose a specific kind of leadership that fosters (team) work engagement as a key (mediating) variable in the motivational process. Recent research has focused on leadership that specifically aims at increasing work engagement—that is, engaging leadership [21,22,23]. Engaging leadership (EL) [21] refers to a positive leadership style that fosters employees’ work engagement through a specific psychological mechanism that can be described using the Self Determination Theory (SDT). As Schaufeli and Taris [24] argued, the JD-R Model is a descriptive model. Hence, when incorporating particular resources and outcomes to this model, additional explanatory psychological theories are needed, such as, in this case, SDT. Engaging leaders promote the fulfilment of employees’ basic needs for competence, relatedness, and autonomy by strengthening, connecting, empowering and inspiring employees, and hence, increase their follower’s levels of work engagement [21].

Scholars stressed that the study of leadership is inherently multi-level in nature [25] (p.4). Furthermore, team member’s perceptions of engaged leadership converge, meaning that team members generally agree about the level of engaging leadership of their supervisor [26,27]. Thus, in the present research, the impact of team-level EL will be examined on job outcomes, as mediated by (team) work engagement. At the team level, the Input–Mediator–Output–Input (IMOI) team effectiveness framework [28] will be used, particularly for introducing team work engagement as a mediator. More specifically, we propose that leaders who display an engaging leadership style, collectively regarding their entire team as well individually regarding each of the team members, may effectively foster team work engagement and individual work engagement. This, in turn, fosters positive outcomes at the team and individual level, respectively. 

Three crucial job outcomes for organizations to survive and be competitive are measured, namely performance, learning, and innovation—both at the team and individual level. Organizational studies show that these three outcomes are positively interrelated [29,30]. To test our model of how engaging leadership at the team level is beneficial for job outcomes both at the individual and team level via TWE and WE, we conducted a two-wave multi-level study.

In sum, our study offers three contributions to the literature. First, by testing the cross-level effects of team-level leadership on individual job performance. By uncovering the mediating role of TWE and WE, this study gives insight in how team-level EL impacts team and individual job outcomes and, hence, features JD-R Model at the team level. Research to date has mainly focused on engagement at the individual level, whereas at the role of team-level TWE is under-researched.

Second, the novel concept of engaging leadership that explicitly focuses on increasing WE is introduced as an antecedent, which, via WE and TWE, impacts job outcomes at the individual and team level. Contributing to leadership literature, the basic tenet of EL is that engaging leaders behave in such a way that they fulfill employees’ work-related basic needs, which, in their turn foster work engagement among their employees. Moreover, we examine EL at the team level, emphasizing its multi-level nature that so far has been studied rather scarcely. 

**Figure 1:**
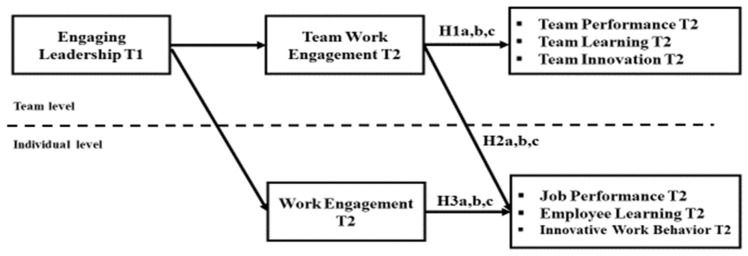
Research Model of Engaging Leadership, Work Engagement, and Job Performance Relationship at the Individual and Team Level.

Third, our study proposes a mediation model that explores the relationships between team-level EL, engagement and job outcomes at the individual and team level, as they evolve over a one-year period. Hence, we consider the complex and dynamic relationships over time between team leadership on the one hand and team and individual outcomes on the other hand, as mediated by work engagement—both at the team and individual level (see Figure 1).

## 2. Theoretical Review and Hypothesis Development

### 2.1. The Concept of Engaging Leadership

The concept of engaging leadership [21] assumes that engaging leaders behave in such a way that they fulfill their followers’ work-related basic needs. Particularly, SDT postulates that employees are likely to show high levels of energy, concentration, and persistence to the degree that their needs for autonomy, competence, and relatedness are satisfied [31]. In other words, when the follower’s basic needs are satisfied, they are likely to feel more engaged in terms of vigor, dedication, and absorption. 

Schaufeli [21] argued that engaging leaders fulfill the basic psychological needs of their employees by performing certain leadership behaviors, namely strengthening, empowering, connecting, and inspiring. When employees are strengthened, because their supervisor delegates responsible and challenging tasks, they will feel more competent after task completion (“Yes, I can”). When employees relate to others in their team, since their supervisor encourages close collaboration and interpersonal bonding, they will feel a strong sense of belongingness (“I feel at ease in my team”). When employees are empowered, as their supervisor encourages their voice and recognizes their ownership, they will feel autonomous (“I can make my own decisions”). Finally, when employees are inspired by their supervisor to contribute personally to an important overall goal this will increase their meaningfulness (“I can make a significant contribution”). 

Although the need for meaningfulness has not been identified as a separate basic need by SDT, theoretical and empirical arguments have been proposed in its favor [32,33]). SDT views meaningfulness as an outcome of basic psychological need satisfaction rather than as a specific need [34] (pp. 252–254). However, following Baumeister [35] and Frankl [36], we believe that the need for meaningfulness, which is defined as the desire to be engaged in activities that are useful, important, significant, and is in line with one’s personal values, plays a fundamental role in human motivation. Furthermore, meaningfulness has a strong positive association with work engagement [37]. A recent cross-national study that included samples from Indonesia and Russia found that the satisfaction of basic psychological needs (including the need for meaningfulness) mediated the relationship between engaging leadership and work engagement in both countries [23]. This result supports the assumption that engaging leaders foster their employee’s work engagement through satisfying their basic needs for competence, relatedness, autonomy, and meaningfulness. 

The current study assumes relationships among team-level EL, (team) work engagement, and job outcomes by drawing on the Job Demands–Resources (JD-R) Model [20,38]), Self Determination Theory [39], and the Input–Mediator–Output–Input (IMOI) team effectiveness framework [28]. We concur with Bliese, Halverson, and Schriesheim [25], who argue that leadership should be studied as a multi-level phenomenon, which means to investigate its consequences at the team as well as at the individual level. To date, research on engaging leadership and work engagement has been carried out almost exclusively at the individual level. The current study focuses on team-level EL, meaning that we aggregate the individual perceptions team-members have about their supervisor.

### 2.2. Team-Level Effect of Engaging Leadership at the Team Level on Team Outcomes as Mediated by Team Work Engagement

We set out this study to provide empirical evidence for the mediation of TWE that may explain the relationship between engaging leadership at the team level and team outcomes. Three indicators of team outcomes are included: (1) team performance, which refers to formal team job behaviors (team in-role behavior) as well as to behaviors that exceed what the team expected to do (team extra-role behavior) [40]; (2) team learning, which refers to the ongoing team process of reflection and action, characterized by team members asking questions, seeking feedback, experimenting, reflecting on results, and discussing errors or unexpected outcomes of actions [41]; (3) and team innovation, which refers to the intentional introduction and application within teams of ideas, processes, products or procedures, which are new and benefiting to the team [42] (p. 9).

Based on the motivational process of Job Demands–Resources (JD-R) Model, the more employees can draw upon job resources the more engaged they feel [20]. Furthermore, leaders manage and allocate job demands and resources in such a way as to increase their follower’s levels of (team) work engagement [20]. Although not all job demands and resources are controlled by the leaders, they have a crucial impact on (the allocation of) demands and resources. For example, leaders determine to a large extent the amount of work that employees have to do, and hence their workload. Moreover, the negative effect of emotional and physical demands may be buffered by a high quality, supportive relationship between leaders and followers [20]. Finally, by performing feedback, showing appreciation and recognition, leaders boost this type of job resources even though salary, an example of organizational-level job resources, may be not determined by the leaders. Thus, seen from this perspective, team-level EL may in and of itself act as a resource for teams that would help them increase their team work engagement. For instance, team-level EL may stimulate a supportive team climate, which subsequently, over time, may increase team work engagement. A recent study that included 62 teams from 13 organizations confirmed this reasoning by showing that the relationship between team social resources (e.g., a supportive team climate and proper coordination of tasks) and team performance was mediated by TWE [16].

Similarly, engaging leaders empower and connect their team members so that it makes them feel at ease in the team and they feel safe to express and sharing their ideas, which will stimulate their positive experiences and, hence, increase TWE. Engaging leaders who connect their team members build a pleasant and trusting atmosphere between team members, which makes it easier to communicate ideas about new ways of working and to reduce job demands [43]. As a consequence, the possibility to learn among team members and to be innovative as a team may increase as well. 

Drawing from the Input–Mediator–Output–Input (IMOI) team effectiveness framework [28], it is assumed that to be effective and perform, teams need to be well-functioning. In the current research, the positive affective-motivational state of team work engagement plays a crucial role as a mediator, which enable teams to function well. Previous research supported the positive relationship between collective engagement or TWE and positive job outcomes, such as team performance [16,44], team satisfaction [45], and perceived collective efficacy and subjective wellbeing [19]. 

In the current study, team-level EL acts as team-level input, and team performance, team learning, and team innovation as outputs. Meaning that engaging leaders provide team resources, including a positive team spirit and supportive team climate, and collective enthusiasm, and team efficacy (input). As a result, the team feels energetic, dedicated and is focused, i.e., collectively engaged (mediator), which, in turn, leads to a greater discretionary team effort in terms of team performance, team learning, and team innovation (output). Hence, we formulate:

**Hypothesis** **1.** *Team work engagement at T2 mediates the relationship between engaging leadership at the team level at T1 and T2 team performance (H1a), team learning behavior (H1b), and team innovation (H1c).*

### 2.3. Cross-Level Effect of Engaging Leadership at the Team Level on Job Outcomes as Mediated by Team Work Engagement

The current study assumes cross-level relationships between engaging leadership at the team level and team work engagement on the one hand and individual job outcomes (i.e., job performance, employee learning, and innovative work behavior) on the other. Job performance refers to activities that are related to the formal job (in-role behavior) as well as to activities that go beyond the formal job description (extra-role behavior), while team performance refers to team-in-role and extra-role behaviors [40]. Employee learning is seen as an ongoing process of reflection and action, characterized by asking questions, seeking feedback, experimenting, reflecting on results, and discussing errors or unexpected outcomes of actions [41]. Finally, innovative work behavior refers to complex work behaviors that including idea generation, idea promotion, and idea realization [46]. 

We argue that team-level EL and TWE may increase individual job outcomes by drawing on JD-R Model and an emotional contagion mechanism. Later, in the measurement section, we will explain how we aggregated the individually perceived TWE at the team level. The underlying process that is involved is known as emotional contagion. Bakker, van Emmerik and Euwema [47] identified emotional contagion as a crucial crossover mechanism that leads to the emergence of a shared psychological state, such as team work engagement. Hatfield, Cacioppo, and Rapson [48] define emotional contagion as: “the tendency to automatically mimic and synchronize facial expressions, vocalizations, postures, and movements with those of another person’s and, consequently, to converge emotionally” (p. 5). Emotions can spread among individuals. Thus, different people can share and express the same emotional state [49], which can either be positive such as engagement or negative such as burnout [50]. 

When engaging leaders inspire their team, by installing high expectations, connecting the team goal with more overall meaningful (organizational) goals, thereby conveying an optimistic vision for the future, this might induce positive affect and increase team motivation in terms of TWE. Furthermore, this collective work engagement increases collective efficacy beliefs [51] as well as individual-level work engagement [50]. That is, when employees experience that they are part of an engaged team (which is vigorous, dedicated and absorbed), they are more likely to feel engaged at the individual level (and vice versa). As a result, they will increase their willingness to put more effort into their jobs. In the context of the present study, emotional contagion means that team members synchronize their facial expressions, vocalizations, postures, movements, and so on with those of others in the team who feel engaged so that—as a result—they also feel engaged themselves. In line with this reasoning Bakker, Albrecht, and Leiter [52] showed that team engagement influences individual employee performance through individual-level engagement. 

As leaders have a special role in fostering work engagement among their followers [53], team-level EL fosters a shared feeling of engagement in the entire team and fosters a positive psychological climate that eventually motivates individual team members to perform, learn and innovate. For instance, it has been found among students that team-based learning increased when they receive more feedback (competence support), collaborated more closely (relatedness support), and had greater responsibility (autonomy support) [54,55].

Unsworth and Clegg [56] suggested that besides motivation and autonomy, a positive team climate will help team members to feel free to voice new ideas and feel confident that the group will support these. In other words, team members are more likely to make efforts to innovate when they expect positive responses from other team members. Team-level EL promotes TWE of the entire team, which results in a positive, supportive climate that stimulates individual team members to voice their innovative ideas. Indeed, Scott and Bruce [57] found that a supportive team climate led to more innovation outcomes. Hence, we formulate:

**Hypothesis** **2.** *Team work engagement at T2 mediates the relationship between engaging leadership at the team level at T1 and T2 job performance (H2a), employee learning (H2b), and innovative work behavior (H2c).*

### 2.4. Cross-Level Effect of Engaging Leadership at the Team Level on Job Outcomes as Mediated by Individual Work Engagement

Work engagement has a positive impact on positive job-related attitudes, health and wellbeing, extra-role behavior, and job performance [14,58,59,60]. Moreover, engaged employees exhibit personal initiative and have a strong motivation to learn [14]. Employees who feel engaged are intrinsically motivated [14], proactive [58], more creative [11,61], and display innovative behaviors at work [9,10].

Drawing upon the JD-R Model [20] and Self Determination Theory [39], engaging leadership was found to be a specific leadership behavior that increased work engagement by providing more job resources [21,22]. Engaging leaders who inspire, strengthen and connect employees enable them to perceive and use more resources in their work environment such as social support from colleagues and job autonomy [22]. In line with this reasoning, a recent study found that engaging leadership increases work engagement by fulfilling employees’ basic psychological needs for autonomy, competence, relatedness, and meaningfulness [23].

Team-level EL that increases employees’ feeling of being competent, autonomous, empowered and inspired enables employees to improve their work and proactively find new ways to make their work even more appealing for them. When employees perceive their job as valuable, meaningful and motivating, they tend to show more interests in their daily work, which motivates them to explore better ways of doing their jobs [62]. Letting their employees use their own strengths, practicing their competences, and exercising their self-initiative will fosters employees’ work motivation. While working on complex tasks, highly motivated employees showed more innovative work behaviors than less motivated employees [63]. Previous research supported the idea that intrinsic motivation, which is akin to engagement, plays a mediating role between psychological contract and innovative work behavior [9], and between learning organization and innovative work behavior [64]. When the perceptions of engaging leadership are shared in the team, engaging leaders may successfully stimulate a positive and supportive team climate that fosters employees’ WE because they can draw upon more resources and their basic needs are satisfied. In turn, engaged employees may produce positive job outcomes by putting greater effort into accomplishing, learning and exploring their jobs. Hence, we hypothesize that:

**Hypothesis** **3.** *Individual work engagement at T2 mediates the relationship between engaging leadership at the team level at T1 and T2 job performance (H3a), employee learning (H3b), and innovative work behavior (H3c).*

## 3. Method

### 3.1. Sample and Procedure

#### 3.1.1. Sample

The current research was conducted in an Indonesian agriculture state-owned company, which mainly produces crude palm oil and other agricultural commodities, such as tea, cacao, rubber, and vegetables. The company includes over 26,000 employees working at various regions on the island of Northern Sumatra. We included all 8 districts of 9 districts (39 out of 41 total plantations), except one district with 2 plantations in another province. Each district consists of several plantations, and each plantation consists of several units depending on the size of the district and the plantation. Employees working in the same unit under the same supervisor were considered a team. Conveniently selected, mostly because of the closer location to the head office in the capital of North Sumatra province, we included 100 units of 8 districts and each unit consist of at least 7 team members. In total, 700 paper-and-pencil surveys contained 9 study variables bundled in a book format were prepared and handed out to about 700 employees. 

The data used were collected in two waves: in March–June 2017 and one year later in March–July 2018. In total, 607 employees participated (response rate 87.3 percent), nested in 94 teams, with team sizes ranging from 6 to 10 employees. In the second wave, 435 employees participated (response rate 71 percent), nested in 75 teams, with team sizes ranging from 6 to 8 employees. From these 435 employees, 27 participants were excluded due to incomplete data, 84 participants dropped out because they had their supervisor changed, 34 participants were dropped as they moved to another team, and 66 participants were the supervisors and, thus, they were excluded as well. Hence, the final longitudinal sample consisted of 224 employees, nested in 54 teams. All participants were males, working as blue-collar workers, which means they do different jobs on the plantations, such as land preparing, planting, harvesting, transporting, processing, and marketing. Some characteristics of the sample are presented in Table 1.

#### 3.1.2. Procedure

We collected the data with the official agreement from the company. Research assistants handed the surveys in sealed envelopes to each of the participants individually. The surveys were completed during working hours and the completed surveys were sent back within two weeks in a sealed envelope to the research assistants collectively per team via a distribution officer. Participants received a written description of the study along with an informed consent together with the survey. In this description, it was announced that the same data will be collected in the next year. The anonymity of the data was emphasized, and we used individually assigned codes to link the data of both waves. For the second wave, surveys were handed out using a similar procedure collected the same data of 9 study variables.

### 3.2. Measurements

Self-reported five-point Likert frequency scale ranging from 1 (never) to 5 (always) were used, except for the job performance and team performance scales, that ranged from 1 (completely disagree) to 5 (completely agree). All items were translated from English into Bahasa Indonesia following the double translation procedure [65]. 

Engaging leadership was measured by the 12 items of the Engaging Leadership Scale [21,23], which assesses four aspects of engaging leadership with three items each; strengthening, connecting, empowering, and inspiring. Sample items are: “My supervisor delegates tasks and responsibilities to team members” (strengthening); “My supervisor encourages collaboration among team members” (connecting); “My supervisor gives team members freedom to complete their tasks” (empowering), and; “My supervisor is able to enthuse team members with his plans” (inspiring). The values of Cronbach’s alpha for the total scale at T1 and T2 were 0.86 and 0.86, respectively.

Work engagement was assessed with the 9 item version of the Utrecht Work Engagement Scale (UWES) [66]. Previous studies carried out in other countries have shown that the UWES has satisfactory psychometric properties [66,67]. The UWES assesses three aspects of work engagement, namely vigor, dedication, and absorption. Sample items are: “At my work, I feel bursting with energy” (vigor), “I am proud of the work that I do” (dedication), and, “I get carried away when I’m working” (absorption). The values of Cronbach’s alpha for this scale at T1 and T2 were 0.87 and 0.86, respectively.

Team work engagement was assessed with the 9 item version of the Team Work Engagement Scale [18]. Following Costa, Passos, and Bakker [18], in the current study, we used a reference shift from “I/me” to “we/our” in all questionnaire items to assess TWE. Like the UWES, the TWE scale consists of three aspects: team vigor, team dedication, and team absorption. Sample items are: “At our job, we feel strong and vigorous” (vigor), “We are proud of the work that we do” (dedication), and “We are immersed in our work” (absorption). The values of Cronbach’s alpha for the total scale at T1 and T2 were 0.87 and 0.87, respectively.

Team performance was measured by the Team Performance Scale by Torrente, Salanova, Llorens, and Schaufeli [16] adapted from Goodman and Svyantek [40], which assesses team in-role behavior and extra-role behavior with three items each. Sample items are: “My team achieves its work goals” (in-role behavior), “We perform roles that are not formally required but which improve the organization’s reputation” (extra-role behavior). The values of Cronbach’s alpha for the total scale at T1 and T2 were 0.82 and 0.79, respectively.

Team learning behavior was measured by the 7 item Team Learning Behavior Scale by Edmonson [41]. A sample item of team learning behavior is “My team frequently seeks new information that leads us to make important changes”. The values of Cronbach’s alpha for this scale at T1 and T2 were 0.76 and 0.80, respectively.

Team innovation was examined with the 4 item Team Innovation Scale by Drach-Zahavy and Someh [68], adapted from West and Wallace [69]. A sample item of team innovation is “The team initiated new procedures and methods”. The values of Cronbach’s alpha for this scale at T1 and T2 were 0.87 and 0.84, respectively.

Individual job performance was measured by 8 items of the Job Performance Scale, including four items tapping in-role behavior and four items tapping extra-role behavior component [40]. Sample items are: “I fulfill all requirement of my job” (in-role behavior), and, “I volunteer to do things that are not formally required by my job” (extra-role behavior). The values of Cronbach’s alpha for this scale at T1 and T2 were 0.80 and 0.79, respectively.

Employee learning was measured by the Employee Learning Scale with 6 items from the 7 item learning behavior scale [41]. Consistent with the level of analysis, which was at the individual level, the referent was changed from “My team” to “I”. Based on our CFA, item number 2 (“I tend to handle differences of opinion privately or off-line, rather than addressing them directly as a group”) has a very low factor loading, and we decided to remove it due to the cultural reasons. To maintain group harmony, Indonesians tend to avoid direct communication in handling interpersonal conflicts, which makes them feel uncomfortable. A sample item of the employee learning scale was “I frequently seek new information that leads me to make important changes”. The value of Cronbach’s alpha for this scale at T1 and T2 were 0.77 and 0.77, respectively.

Innovative work behavior was examined with the 4 item Innovative Work Behavior Scale [46]. It consists of 3 aspects namely, generating ideas, promoting ideas, and applying ideas with items like “I generate original solutions for problems”, “I acquire approval for innovative ideas”, and “I transform innovative ideas into useful applications”. The values of Cronbach’s alpha for this scale at T1 and T2 were 0.92 and 0.92, respectively.

### 3.3. Preliminary Analysis

#### 3.3.1. Confirmatory Factor Analysis

To test the validity of the nine-factor measurement model, a multi-level confirmatory factor analysis (MCFA) was conducted, a technique that may also be used to account for group-level influences when verifying individual levels [70]. The result showed that the hypothesized nine-factor model had a good fit to the data (χ2 (231) = 367.748, *p* < 0.001, Root Mean Square Error of Approximation (RMSEA) = 0.051, Standardized Root Mean Square Residual (SRMR)Within = 0.045, Comparative Fit Index (CFI) = 0.942, Tucker Lewis Index (TLI) = 0.930.

#### 3.3.2. Data Aggregation

Although some of the variables will be employed for the team-level analysis and we used the collective referents such as ‘My team’ and “We”, we measured these at the individual level. Thus, before continuing our analyses it should be checked whether the aggregation individual scores into a mean score for each team, is feasible [71]. To justify aggregation, the within-group agreement (Rwg) [72], the reliability of a single assessment of the group mean or Intraclass Correlation (ICC1) [73], and the F-test (indication whether average scores differed significantly across teams) were calculated. The aggregation statistics for individual-level variables are presented in Table 2.

For engaging leadership, for example, the Rwg ranged from 0.78 to 1.00, with 98% of the teams meeting the criterion of 0.70 [74]; ICC1 = 0.10, F = 1.586, *p* = 0.014, meaning that 98% of team members agreed in their leader ratings to the extent that the perception can be perceived as shared. Furthermore, 10 percent of the variance in engaging leadership can be explained by the grouping effect [73]. As the within-group agreement for all variables ranged from 0.71 to 1.0 across teams, and thus reached the required minimum in each team, aggregating all variables to a team-level construct was deemed feasible [72]. Moreover, according to Dyer, Hanges, and Hall [70], ICC(1)’s tend to range between 0.00 and 0.50 with a median value of 0.12. Thus, the relatively low ICC1 (0.10) for engaging leadership could be aggregated as well. When applying ICC(1), if the between-group F-test from the ANOVA is significant, aggregation of participants within each group is considered justified [75], as the variance between the groups is not caused by measurement error. 

To conclude, based on our results, it was found that team membership explained a considerable amount of variance in individual ratings of engaging leadership, team work engagement, team performance, team learning behavior, and team innovation. However, for team learning and team innovation, some caution is warranted since in the former case only 65% of the teams meet the criterion of 0.70, and in the latter case, the F test was nonsignificant. Nevertheless, we included these two variables in our further analysis following LeBreton and Senter [74], who recommended to aggregate groups as long as they have satisfactory Rwg values.

### 3.4. Strategy of Data Analysis

Random coefficient analyses [76], the robust maximum likelihood estimator, HLM software, SPSS software, and R software were used to test our hypotheses. First, ordinary least square (OLS) regression-based analysis was conducted using SPSS to test the T1 engaging leadership at the team level on each of T2 team outcomes mediated by T2 TWE (H1a,b,c). Second, a multi-level analysis was performed using HLM. We simultaneously tested the direct and mediated cross-level effects of T1 engaging leadership at the team level on each of T2 individual job outcomes, as mediated by T2 TWE and WE (H2a,b,c and H3a,b,c). Finally, a Monte Carlo test was performed using R to check the robustness of the results [77,78].

## 4. Results

### 4.1. Descriptive Statistics

Table 3 shows the means, standard deviations, Cronbach’s α, and correlations of the study variables.

### 4.2. Hypotheses Testing

Hypothesis 1 stated that the effect of T1 engaging leadership at the team level on T2 team outcomes is mediated by T2 team work engagement. OLS regression revealed that there was no significant relationship between T1 engaging leadership at the team level and T2 team performance as mediated by team work engagement at T2 (β = 0.25, *p* = 0.07). Nevertheless, there was a significant direct relationship between engaging leadership at the team level and team performance T2 (β = 0.47, *p* < 0.01). Thus, Hypothesis 1a was not supported. 

OLS regression revealed a significant relationship between T1 engaging leadership at the team level and T2 team learning, as mediated by T2 team work engagement (β = 0.47, *p* < 0.01); at the same time, there was no significant direct relationship between engaging leadership at the team level and team performance T2 (β = 0.08, *p* = 0.58). Thus, Hypothesis 1b was supported; T2 team work engagement fully mediated the relationship between T1 engaging leadership and T2 team learning. 

OLS regression revealed a significant relationship between T1 engaging leadership at the team level and T2 team innovation, as mediated by T2 team work engagement (β = 0.27, *p* < 0.05). Simultaneously, there was a positive significant relationship between T1 engaging leadership at the team level and T2 team innovation (β = 0.40, *p* < 0.01). Thus, Hypothesis 1c was supported: T2 team work engagement partially mediated the relationship between T1 engaging leadership and T2 team innovation. The result of OLS analyses for H1a,b,c are shown in Table 4.

Hypothesis 2 states that the cross-level effect of T1 engaging leadership at the team level on T2 individual-level job outcomes (job performance, employee learning, innovative work behavior) is mediated by T2 team work engagement. Hypothesis 3 states that the cross-level effect of T1 engaging leadership at the team level on T2 individual level job outcomes is mediated by T2 work engagement.

HLM results with random slopes yielded a significant mediation effect of T2 team work engagement of the relationship between T1 engaging leadership at the team level and T2 job performance (γ = 0.34, *p* < 0.01). However, no significant mediation effect was found of T2 team work engagement of the relationship between T1 engaging leadership at the team level and T2 employee learning (γ = 0.34, *p* > 0.05), and also no significant mediation effect was found of T2 team work engagement of the relationship between T1 engaging leadership at the team level and T2 innovative work behavior (γ = 0.49, *p* > 0.05). Thus, only hypothesis 2a was confirmed; the relationship between T1 engaging leadership at the team level and T2 individual job performance was partially mediated by T2 team work engagement. Hypotheses 2b and 2c that referred to employee learning and employee innovative work behavior, respectively, were not supported.

HLM with random slopes yielded a significant positive effect of T1 engaging leadership at the team level on T2 job performance (γ = 0.32, *p* < 0.01), T2 employee learning (γ = 0.83, *p* < 0.01), and T2 innovative work behavior (γ = 0.83, *p* < 0.01) respectively. In addition, HLM results (see Table 5) also showed a significant mediation effect of T2 work engagement on the relationship between engaging leadership at the team level and T2 job performance T2 (γ = 0.37, *p* < 0.01), T2 employee learning (γ = 0.60, *p* < 0.01), and T2 innovative work behavior T2 (γ = 0.76, *p* < 0.01). Thus, hypotheses 3a–c were supported. HLM results with random slopes for H2a,b,c and H3a,b,c are shown in Table 5. 

To test the robustness of the mediating effects of work engagement and team work engagement, a Monte Carlo test [79] was performed using R. A summary of all results is shown in Table 6.

The result of this Monte Carlo test for job performance variable indicated significant cross-level mediating effects of T2 individual work engagement (Effect = 0.22, 95%CI = [0.05, 0.33]) and T2 team work engagement (Effect = 0.17, 95%CI = [0.01, 0.49]) on the relationship between T1 engaging leadership at the team level and T2 individual job performance. However, no significant mediating effect of T2 team work engagement was observed (Effect = 0.14, 95%CI = [−0.02, 0.33]) for the relationship between T1 engaging leadership at the team level and T2 team performance.

Furthermore, the result for team learning and employee learning were significant. This means that the effect of T1 engaging leadership at the team level on T2 team learning and individual learning is mediated by T2 team work engagement (Effect = 0.44, 95%CI = [0.15, 0.82]) and T2 work engagement (Effect = 0.22, 95%CI = [0.08, 0.52]), respectively. However, the cross-level mediating effect of T1 engaging leadership at the team level and T2 individual employee learning via T2 team work engagement was not significant (Effect = 0.28, 95%CI = [−0.01, 0.53]).

Finally, the results for innovative work behavior and team innovation were significant. The effect of T1 engaging leadership at the team level on T2 team innovation and T2 innovative work behavior is mediated by T2 team work engagement (Effect = 0.20, 95%CI = [0.01, 0.49]) and T2 work engagement (Effect = 0.32, 95%CI = [0.11, 0.63]), respectively. However, the cross-level mediating effect of T1 engaging leadership at the team level and T2 innovative work behavior via T2 team work engagement was not significant (Effect = 0.35, 95%CI = [−0.03, 0.75]). 

In sum, these outcomes from the Monte Carlo test supported the previous OLS regression and HLM’s hypotheses testing. Hence, the robustness is confirmed. 

## 5. Discussion

The findings of this study showed at the team level, that engaging leadership at the team level at time 1 was positively related to team learning and team innovation at time 2, through TWE at time 2 (H1b, c). At the cross level, engaging leadership at the team level at time 1 was positively related to individual job performance, employee learning, and innovative work behavior at time 2 through WE at time 2 (H3a,b,c). These results confirmed earlier research on engaging leadership and work engagement [21,22,23], and on work engagement and job outcomes such as job performance, employee learning and innovative work behavior [9,10,14,58,59,60]. However, the cross-level relationship between engaging leadership at the team level at time 1 and individual job outcomes at time 2, through TWE at time 2 was only observed for job performance (H2a), but not for learning and innovative work behavior. Hence, taken together, only three of the nine hypotheses were not confirmed.

### 5.1. Theoretical Contributions

Mediated by team work engagement at time 2, there were significant relations between the team-level effect of engaging leadership at the team level at time 1 and team outcomes at time 2 namely, team learning and team innovation at time 2. However, the link was not significant between engaging leadership at time 1 and team performance at time 2 (H1a). Contrary to previous research on the mediating effect of TWE on team performance [16], part of our first hypothesis (H1a) was not supported, perhaps because of cultural and/or group related reasons.

Indonesian culture has a collectivistic orientation [79], which is illustrated by the fact that most employees in this study have a long tenure; more than 80 percent works in this company for more than 10 years, and almost 50 percent works in the same team for more than 10 years. This long tenure may have contributed to a high level of cohesiveness and conformity in the teams. One of the negative sides of highly-cohesive groups, groupthink, may therefore exist in the work teams. Groupthink may hinder individual team members to voice dissenting opinions, which, in turn, might hinder team performance [80].

Furthermore, mediated by team work engagement at time 2, the relation between the cross-level effect of engaging leadership at the team level and individual job outcomes at time 2 was only significant for job performance. In contrast, the associations with employee learning and innovative work behavior at time 2 (H2b,c) were not significant. Having supportive interactions and a positive climate of TWE among team members, the possibility for employees to share their knowledge and exchange ideas increases. However, when the teams are highly cohesive and interdependent, they perhaps do not consider their effort in learning and innovating as an individual effort (H2b,c were not supported) but more as a collective effort, namely team learning and team innovation (H1b,c were supported). Again, groupthink may play a role in our sample that seems to have a high level of cohesiveness due to socio-cultural factors. Tollefsen [80] suggested that one of the factors promoting groupthink is social cohesion where team members are committed to do something together and are committed to support the joint actions. In our case, individual learning and innovative work behavior may be not seen as the joint actions. Moreover, a highly cohesive team might inhibit team members from initiating discretionary effort in teams, which might foster team performance (H1a was not supported). Yet, the positive climate of a cohesive team worked for individual job performance; that is, TWE stimulates employees to perform better (H2a was supported). 

It seems that the shared positive emotion of team work engagement fosters a positive climate that only works for individual performance but not for team performance. As a matter of fact, the cohesiveness–performance relationship is stronger when cohesiveness is defined in terms of commitment to the group task, rather than as emotional attraction [81]. Thus, in addition to the mediating role of team work engagement in the relationship between leadership–team performance, future research might consider specific team-level moderating variables that may influence the leadership–TWE–team performance relationship, such as group norms and task orientation.

Additionally, even though the current research suggests that organizational environment variables are important to foster employee learning and innovative work behavior, earlier research on innovative behavior was primarily concerned with individual characteristics (e.g., personality and motivation) [64], which may also apply for employee learning. This speculation is supported by the confirmation of hypotheses 3a–c that assumes that the link between engaging leadership at the team level and individual job outcomes is mediated by individual work engagement.

Furthermore, drawing on the Input–Mediator–Output–Input (IMOI) team effectiveness framework [28], engaging leadership at the team level and team work engagement can be included as a promising Input and Mediator in team studies, especially, in predicting team learning and team innovation. Moreover, as suggested by Ilgen, Hollenbeck, Johnson, and Jundt [28], to study the multi-level and dynamic nature of teams, the current research presents the cross-level effect of team-level input to the individual output via team and individual mediator. As an input, engaging leadership at the team level has multiple effects on teams and individual team members. Thus, it acts as a positive antecedent in learning and innovation in a team.

Drawing upon the JD-R Model and SDT, instead of including leadership as a specific antecedent, supervisors’ support was considered as one of the job resources. Nevertheless, leaders play an important role in managing job and organizational resources, which are necessary to achieve individual and team goals. As is illustrated in the current study, engaging leaders increased team and individual job outcomes, via team work engagement and individual work engagement. Thus, the current study extends research on work engagement by integrating engaging leadership in the motivational process of the JD-R Model and examining it at the team level. By integrating findings from individual research of work engagement into the JD-R Model, we introduce the concept of engaging leadership [21,23]. By combining the engaging leadership research model with a two wave multi-level study, our research model increases our understanding of engaging leadership at the team level, as we can now explain how engaging leadership at the team level fosters learning and innovation both at the team level and individual level.

Positive leadership styles such as transformational leadership [82] and authentic leadership [83] have a positive relationship with work engagement. However, none of them specifically focused on fostering work engagement. Recent empirical research supported the idea that engaging leadership focusing on inspiring, strengthening, connecting, and empowering employees increases their levels of work engagement indirectly via the fulfillment of employees’ basic psychological needs for meaning, autonomy, competence and relatedness [23]. In other words, the basic need fulfillment mediates the relation between engaging leadership and followers’ work engagement. Thus, the leadership concept used in the current study answers the call of academia and business to find a narrow leadership construct to foster engagement by introducing a new specific theory based leadership concept, namely engaging leadership [21]. As suggested by Bormann and Rowold [84], to avoid the construct proliferation in leadership studies, the introduction of a new leadership concept is suggested to be related with relevant outcomes, in our case, to increase work engagement. 

### 5.2. Practical Implications

Following Albrecht, Breidahl, and Marty’s suggestion [85], to promote organizational engagement climate, job resources, and work engagement, organizations and human resources management may perform baseline and feedback surveys to determine the extent to which employees perceived engaging behaviors of their leaders. Furthermore, leaders can be trained or coached [86] as engaging leaders that strengthen, empower, connect, and inspire their employees. 

Furthermore, organizations and HR departments could employ team training to improve their team work engagement, since this aspect of team functioning benefited the collective positive climate in teams, and subsequently benefited their team outcomes, such as team learning and team innovation. For example, a team-based intervention to enhance teamwork and staff engagement was successfully tested in a medical unit in an acute care hospital, resulting in positive outcomes [87].

The education can be given to the team members about the fact that their work engagement and performance might depend on their interaction in their teams and their shared perceptions toward their leaders. Having an open positive relationship in teams is necessary not only for the employees individually, but also for the team as a whole. Moreover, engaged employees are encouraged to exhibit their positivity to their teams by sharing good news and expressing enthusiasm to induce collective engagement. 

Determining team and individual outcomes is important in terms of revealing which mechanism is more prominent to boost specific relevant outcomes. Based on our study, individual outcomes are better predicted by individual work engagement, while team outcomes are better predicted by team work engagement. For example, leaders who want to achieve team learning and team innovation should stimulate team work engagement—for instance, by fostering supportive team climate, coordination and teamwork [16]. Leaders who would like to increase employees’ individual learning and innovative work behavior may focus on increasing individual work engagement. Basically, two routes can be followed to achieve this. First, leaders may seek to satisfy those basic psychological needs of employees that are most valued by them [23]. Second, leaders could manage and allocate demands and resources in such a way that their follower’s engagement increases [21]. From a review, Saks and Gruman [88] concluded that a specific job resource will be related to a specific psychological condition, which will eventually be related to a specific type of employee engagement, such as work engagement, task engagement, or team engagement. 

### 5.3. Limitations and Future Research Direction

First, we tested our research model based on one source of data—employees—and with one method only, namely, the self-report survey. However, Spector argues that the problem of common method variance (CMV), a bias that occurs when both predictors and predicted variables stem from the same source, is overstated [89]. Nevertheless, future research is needed to attest our research model using multisource and multimethod data, for example, using a supervisor’s behavior checklist, observation, or peer rating.

Second, the current study also relied on a specific sample collected from one Indonesian holding company in the agricultural industry, which might raise a generalizability concern. Even though data from one organization is better in capturing the organizational specificity (as opposed to snowball methods of data collection), caution is warranted when applying our results to other organizations. Future research is needed to replicate our research model in different settings in terms of companies and industries. Moreover, future research may also be needed to test the research model in other cultures such as Western and African cultures. The Indonesian culture is specifically characterized as collectivistic with a high power distance, which may therefore limit generalization to other cultures. 

Third, we focused on (team) work engagement as a main mediator to clarify the relation of engaging leadership at the team level on job outcomes at the team and individual level. Research on teams about team learning and innovation mostly used self-directed (self-managed) teams with non-routine types of task, such as IT teams or high-tech and health care teams [2,90]. In the present research, we did not control for the team interdependency and the type of task. Future research may replicate our research model using the aforementioned characteristics of the teamwork.

Fourth, future research may link other positive leadership styles (such as transformational leadership, servant leadership, authentic leadership) simultaneously with engaging leadership to work engagement and identify their unique contributions—for example, exploring the unique contributions of engaging and servant leadership’s components in relation to basic need satisfaction [91] and investigating the relative contribution of those components to individual work engagement, as well as team work engagement.

## 6. Conclusions

Rapid changes in the business environment create pressure on organizations to respond timely and effectively and, as a consequence, demand their teams to perform, learn, and innovate continuously. Integrating the current results of work engagement with the JD-R Model and combining these with the recent leadership literature on work engagement, this study contributes to the multi-level explanation of engaging leadership on job outcomes. Over time, engaging leadership at the team level increases (team) work engagement by stimulating a shared positive affect within the teams, which, in turn, fosters job outcomes at the team level (team performances, team learning, and team innovation) and the individual level (job performance, employee learning, and innovative work behavior). 

## Figures and Tables

**Table 1:** Sample Characteristics (Individual level, N = 224, Team level, N = 54).

Individual’s Characteristics		N	Percent
Educational Level	Elementary education	57	25.4
	Secondary education	126	56.3
	Bachelor	39	17.4
	Master	2	0.9
			
Tenure (year)	0–5	24	10.7
	6–10	12	5.4
	11–15	15	6.7
	16–20	42	18.8
	21 and more	131	58.5
			
Working in current team (year)	0–5	98	43.8
	6–10	35	15.6
	11–15	14	6.3
	16–20	27	12.1
	21 and more	50	22.3
			
Working with current	less than a year	72	32.1
supervisor (year)	1–5	146	65.2
	5–10	6	2.7

**Table 2:** Aggregation Statistics for Individual-Level Variables (Individual Level, N = 224, Team Level, N = 54).

Variables	Rwg Cut off > 0.70	% of Meeting the 0.70 Cut off	F	*p*-Value	ICC
Engaging Leadership T1 Team Work Engagement T2 Team Performance T2 Team Learning T2 Team Innovation T2	0.78–1.00	98%	1.586	0.014	0.10
	0.72–1.00	100%	1.967	0.001	0.15
	0.75–1.00	100%	1.910	0.001	0.12
	0.71–1.00	65%	2.040	0.000	0.10
	0.70–1.00	93%	2.170	0.175	0.15

**Table 3:** Mean (M), Standard Deviation (SD), and Correlations of the Study Variables (Individual Level, N = 224, Team Level, N = 54).

**Team-Level Variables**	**Mean**	**SD**	**Variables**				
			**1**	**2**	**3**	**4**	**5**
Engaging leadership at the team level T1 Team work engagement T2 Team performance T2 Team learning T2 Team innovation T2	4.13	0.25					
	4.49	0.33	0.49 **				
	4.28	0.28	0.47 **	0.42 **			
	3.58	0.48	0.31 *	0.51 **	0.23 ns		
	4.23	0.39	0.53 **	0.47 **	0.46 **	0.48 **	
**Cross-Level Variables**	**Mean**	**SD**	**Variables**				
			**1**	**2**	**3**	**4**	**5**
Engaging leadership at the team level T1 Work engagement T2 Job performance T2Employee learning T2 Innovative work behavior T2	4.13	0.25	(0.861)				
	4.61	0.41	0.29 **	(0.874)			
	4.04	0.48	0.29 **	0.36 **	(0.823)		
	3.60	0.79	0.28 **	0.36 **	0.59 **	(0.757)	
	3.66	0.85	0.38 **	0.43 **	0.64 **	0.76 **	(0.874)

Note. The range of the scales for all variables is 1-5. * *p* < 0.05. ** *p* < 0.01. Values of Cronbach’s α at the individual level between parenthesis.

**Table 4:** The Results of Ordinary Least Squares (OLS) Regression of Team Outcomes (N = 54).

	**Team Performance (No Mediating Effect)**		
	**B**	**SD**	**B**
Step 1			
Constant	2.09	0.57	
Engaging Leadership Time 1	0.53	0.14	0.47 **
Step 2			
Constant	1.72	0.59	
Engaging Leadership Time 1	0.39	0.15	0.35 *
Team Work Engagement Time 2	0.21	0.12	0.25 ns
*R* ^2^			0.27 **
*F*			9.43 **
Δ*R*^2^			0.05 ns
Δ*F*			3.32 ns
	**Team Learning (Full Mediation)**		
	**B**	**SD**	**B**
Step 1			
Constant	1.14	1.04	
Engaging Leadership Time 1	0.59	0.25	0.31 *
Step 2			
Constant	−0.53	1.01	
Engaging Leadership Time 1	0.15	0.26	0.08 ns
Team Work Engagement Time 2	0.67	0.20	0.47 **
*R* ^2^			0.27 **
*F*			9.18 **
Δ*R*^2^			0.17 **
Δ*F*			11.74 **
	**Team Innovation (Partial Mediation)**		
	**B**	**SD**	**B**
Step 1			
Constant	0.80	0.75	
Engaging Leadership Time 1	0.83	0.18	0.53 **
Step 2			
Constant	0.25	0.78	
Engaging Leadership Time 1	0.62	0.20	0.40 **
Team Work Engagement Time 2	0.31	0.15	0.27 *
*R* ^2^			0.34 *
*F*			13.17 **
Δ*R*^2^			0.06 *
Δ*F*			4.32 *

** *p* < 0.01; * *p* < 0.05.

**Table 5:** The Hierarchical Linear Modeling Results of the Relationship betweenTeam-Level Engaging Leadership, Individual Level and Team-Level Work Engagement, and Individual- and Team-Level Performance, Learning, and Innovation (Individual Level, N = 224, Team Level, N = 54).

Dependent Variables	WE T2	TWE T2	Job Performance T2		Employee Learning T2		Innovative Work Behavior T2	
	M1	M2	M3	M4	M5	M6	M7	M8
Constant	4.61 (0.03)	0.18 (0.66)	4.04 (0.04)	4.03 (0.04)	3.60 (0.05)	3.60 (0.05)	3.65 (0.07)	3.65 (0.06)
Level 2 (Team Level)								
Engaging Leadership T1	0.46 ** (0.15)	0.65 ** (0.16)	0.57 ** (0.16)	0.32 ** (0.15)	1.06 ** (0.25)	0.83 ** (0.27)	1.16 ** (0.34)	0.83 * (0.40)
TWE T2				0.34 ** (0.16)		0.34 (0.19)		0.49 (0.28)
Level 1 (Individual Level)								
WE T2				0.37 ** (0.09)		0.60 ** (0.14)		0.76 ** (0.14)
R1^2^	0.07		0.08	0.18	0.10	0.16	0.11	0.19
R2^2^	0.21		0.21	0.20	0.33	0.34 **	0.27	0.30
Pseudo R^2^		0.24						
F		16.58 **						

Level 1 = individual level, N = 224. Level 2 = team level, N = 54 (224 participants nested in 54 teams). Unstandardized multi-level modeling coefficients (γ) are shown. Robust standard errors are in parentheses. R1^2^ = individual level variance component, and R2^2^ = team-level variance component, Pseudo R^2^ = proportion of variance explained in dependent variable by predictors at both the team- and individual-levels. ** *p* < 0.01; * *p* < 0.05.

**Table 6:** The Summary of Team-Level and Cross-Level Direct and Mediated Effects of Engaging Leadership at the Team Level on Team-Level and Individual-Level Outcomes (Individual Level, N = 224, Team Level, N = 54).

Path	Effect	SE	LLCI 95%	ULCI 95%
**Test of team-level direct effects (2-2 model)**				
Engaging Leadership T1 > Team Performance T2	0.39 **	0.15	0.09	0.68
Engaging Leadership T1 > Team Learning Behavior T2	0.15 ns	0.26	−0.35	0.67
Engaging Leadership T1 > Team Innovation T2	0.62 **	0.20	0.23	1.01
**Test of cross-level direct effects (2-1 model)**				
Engaging Leadership T1 > Job Performance T2	0.32 **	0.15	0.03	0.62
Engaging Leadership T1 > Employee Learning T2	0.83 **	0.27	0.31	1.36
Engaging Leadership T1 > Innovative Work Behavior T2	0.83 **	0.40	0.04	1.61
**Test of team-level mediated effects (2-2-2 model)**				
Engaging Leadership T1 > Team Work Engagement T2 > Team Performance T2	*0.14 ns*	0.09	−0.02	0.33
Engaging Leadership T1 > Team Work Engagement T2 > Team Learning Behavior T2	0.44 **	0.17	0.15	0.82
Engaging Leadership T1 > Team Work Engagement T2 > Team Innovation T2	0.20 *	0.11	0.01	0.49
**Test of cross-level mediated effects (2-2-1 model)**				
Engaging Leadership T1 > Team Work Engagement T2 > Job Performance T2	0.17 *	0.07	0.01	0.49
Engaging Leadership T1 > Team Work Engagement T2 > Employee Learning T2	*0.28 ns*	0.11	−0.01	0.53
Engaging Leadership T1 > Team Work Engagement T2 > Innovative Work Behavior T2	*0.35 ns*	0.13	−0.03	0.75
**Test of cross-level mediated effects (2-1-1 model)**				
Engaging Leadership T1 > Work Engagement T2 > Job Performance T2	0.22 *	0.12	0.05	0.33
Engaging Leadership T1 > Work Engagement T2 > Employee Learning T2	0.22 *	0.14	0.08	0.52
Engaging Leadership T1 > Work Engagement T2 > Innovative Work Behavior T2	0.32 **	0.20	0.11	0.63

*p < 0.05. **p < 0.01.
